# A transcription-dependent increase in miniature EPSC frequency accompanies late-phase plasticity in cultured hippocampal neurons

**DOI:** 10.1186/1471-2202-10-124

**Published:** 2009-09-29

**Authors:** J Simon Wiegert, Frank Hofmann, Hilmar Bading, C Peter Bengtson

**Affiliations:** 1 Department of Neurobiology, Interdisciplinary Center for Neurosciences, Im Neuenheimer Feld 364, 69120 Heidelberg, Germany; 2 Friedrich Miescher Institute for Biomedical Research, Maulbeerstr. 66, WRO-1066.4.10, CH-4058 Basel, Switzerland; 3 Multi Channel Systems MCS GmbH, Aspenhaustrasse 21, 72770 Reutlingen, Germany

## Abstract

**Background:**

The magnitude and longevity of synaptic activity-induced changes in synaptic efficacy is quantified by measuring evoked responses whose potentiation requires gene transcription to persist for more than 2-3 hours. While miniature EPSCs (mEPSCs) are also increased in amplitude and/or frequency during long-term potentiation (LTP), it is not known how long such changes persist or whether gene transcription is required.

**Results:**

We use whole-cell patch clamp recordings from dissociated hippocampal cultures to characterise for the first time the persistence and transcription dependency of mEPSC upregulation during synaptic potentiation. The persistence of recurrent action potential bursting in these cultures is transcription-, translation- and NMDA receptor-dependent thus providing an accessible model for long-lasting plasticity. Blockade of GABA_A_-receptors with bicuculline for 15 minutes induced action potential bursting in all neurons and was maintained in 50-60% of neurons for more than 6 hours. Throughout this period, the frequency but neither the amplitude of mEPSCs nor whole-cell AMPA currents was markedly increased. The transcription blocker actinomycin D abrogated, within 2 hours of burst induction, both action potential bursting and the increase in mEPSCs. Reversible blockade of action potentials during, but not after this 2 hour transcription period suppressed the increase in mEPSC frequency and the recovery of burst activity at a time point 6 hours after induction.

**Conclusion:**

These results indicate that increased mEPSC frequency persists well beyond the 2 hour transcription-independent phase of plasticity in this model. This long-lasting mEPSC upregulation is transcription-dependent and requires ongoing action potential activity during the initial 2 hour period but not thereafter. Thus mEPSC upregulation may underlie the long term, transcription-dependent persistence of action potential bursting. This provides mechanistic insight to link gene candidates already identified by gene chip analysis to long lasting plasticity in this in vitro model.

## Background

Synaptic plasticity plays an important role in many aspects of brain function such as learning, memory and development [1,2]. Synaptic activity-induced changes in synaptic efficacy can persist for hours, days or even months [3,4] given gene-transcription and subsequent synthesis of new protein [5-7]. Recently, gene chip analysis has identified large numbers of genes regulated by synaptic plasticity [8-10]. The expression and synaptic localisation of activity-induced mRNAs which encode proteins such as Arc/Arg3.1 have been shown to be crucial for the regulation of synaptic plasticity [11,12]. Although numerous gene candidates are emerging, detailed knowledge is still lacking about the synaptic mechanisms affected by the gene expression which transforms early-phase LTP (E-LTP, lasting < 2 h, also known as LTP1) into late-phase LTP (L-LTP, lasting > 2 h, also known as LTP3). While altered pre- and post-synaptic function in E-LTP has been demonstrated with quantal analysis of patch clamp recordings from acute hippocampal brain slices [13-16], little is known about the nature, time course and mechanism of these changes in synaptically induced L-LTP [17]. This lack arises in part from the difficulty of repeating measurements over more than 2 hours from a subset of synapses on the same cell in an acute brain slice.

Here we assess the persistence and transcription dependency of potentiated synaptic transmission using a between cell analysis of brief recordings at multiple time points after its induction. We use a culture based system of synaptically activated long-term plasticity where gene profiling has identified candidates for transcription-dependent late-phase plasticity [10,18]. Removal of inhibitory synaptic transmission in dissociated hippocampal cultures with the GABA_A_-receptor antagonist bicuculline for 15 minutes induces synchronous action potential bursting (AP bursting) which persists for more than 24 hours. This potentiated state in culture mimics that of NMDA receptor-dependent L-LTP in slices, requiring extracellular signal-regulated kinase 1/2 (ERK1/2) signalling and both gene transcription and translation. Neurons in bicuculline treated cultures show an increased frequency of AMPA receptor-mediated mEPSCs at a time point 30 minutes after washout [18]. mEPSCs represent the postsynaptic response to spontaneous release of single neurotransmitter vesicles at functional synapses and thus provide an index of synaptic efficacy and connectivity at a quantal level. mEPSCs are known to be increased in frequency and/or amplitude in LTP in hippocampal slices [19,20]; however it is not known whether such changes persist during L-LTP.

We show that synaptic potentiation, manifested as an increased mEPSC frequency in the hippocampal culture model of LTP, is preserved at transcription-relevant time points (2-6 hours) and requires transcription. This finding points to a common transcription-dependent mechanism in the maintenance of synaptic potentiation and recurrent network bursting which is established within the first two hours after LTP induction.

## Results

### Assessment of AP bursting and synaptic transmission

**Figure 1:**
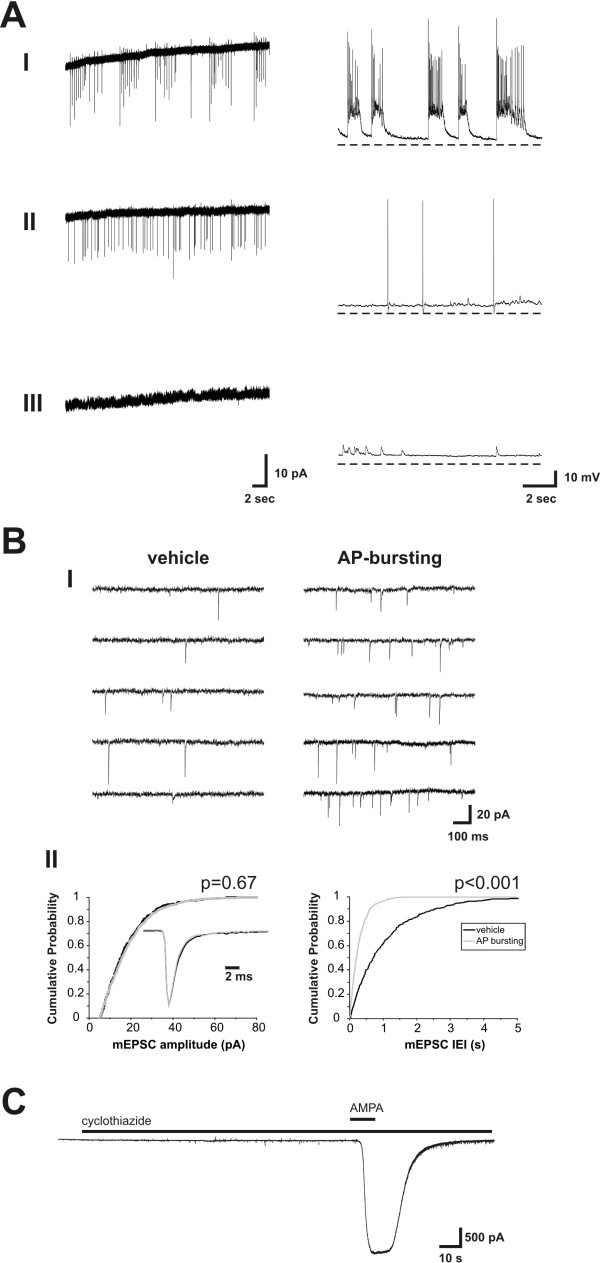
**The assessment of bursting and measurement of mEPSCs**. **(A) **Shown are representative recordings in cell-attached (left) and whole-cell current clamp (right) configurations from neurons showing (**i**) bursting, (**ii**) random activity, and (**iii**) no activity. The slow upward drift of cell-attached recordings is due to the slow improvement of the seal with time. Dashed lines in whole-cell recordings represent -70 mV. **(B) (i) **Traces show mEPSCs recorded in the presence of TTX (1 μM) from neurons one hour after a 15 minute treatment with vehicle (left) or bicuculline (50 μM, right). **(ii) **Cumulative probability histograms generated from these recordings show the distribution of amplitude (left) and inter event interval (IEI, right) of mEPSC events. The inset shows the average of all mEPSCs, normalised for amplitude, in each cell. P values indicate results of Kolmogorov-Smirnoff tests. **(C) **This recording shows a typical response to bath applied AMPA (10 μM) recorded after 2 minutes pre-treatment with cyclothiazide (20 μM).

In cultured hippocampal networks, it has previously been shown that AMPA receptor-mediated mEPSCs show an increased frequency but not amplitude at a time point 30 minutes after LTP induction with a 15 minute bicuculline treatment [18]. To examine the longevity of this upregulated quantal synaptic transmission, we randomly selected one neuron per coverslip and made whole-cell patch clamp recordings of mEPSCs at time points up to 10 hours after treatment with either bicuculline (n = 70 neurons) or vehicle (n = 62 neurons) for 15 minutes. Prior to mEPSC-recordings, activity was assessed in all neurons by both cell-attached and whole-cell current clamp (IC) recordings (Figure 1A). Neurons showed distinct activity patterns categorised as no activity, random activity or recurrent bursting. Bursts were composed of clusters of 5 to 30 spikes separated by relatively spike-free intervals of 5 to 50 seconds [for a full description of AP bursting see 18] (Figure 1A). Cumulative probability histograms for inter event intervals (IEI, 1/frequency) and amplitudes were generated from at least 300 mEPSCs recorded from each cell (Figure 1B). mEPSCs were sensitive to 10 μM DNQX (n = 6) confirming their identity as AMPA receptor-mediated mEPSCs (data not shown). Responses were also recorded to bath applied AMPA (10 μM, 6 ml/min) in the presence of cyclothiazide (20 μM) to prevent desensitisation (Figure 1C). This produced a steady state plateau within 15 s whose amplitude was used as an indication of the total number of functional AMPA receptors per cell.

### Potentiation in synaptic transmission over a 10 hour period following the induction of AP bursting

**Figure 2:**
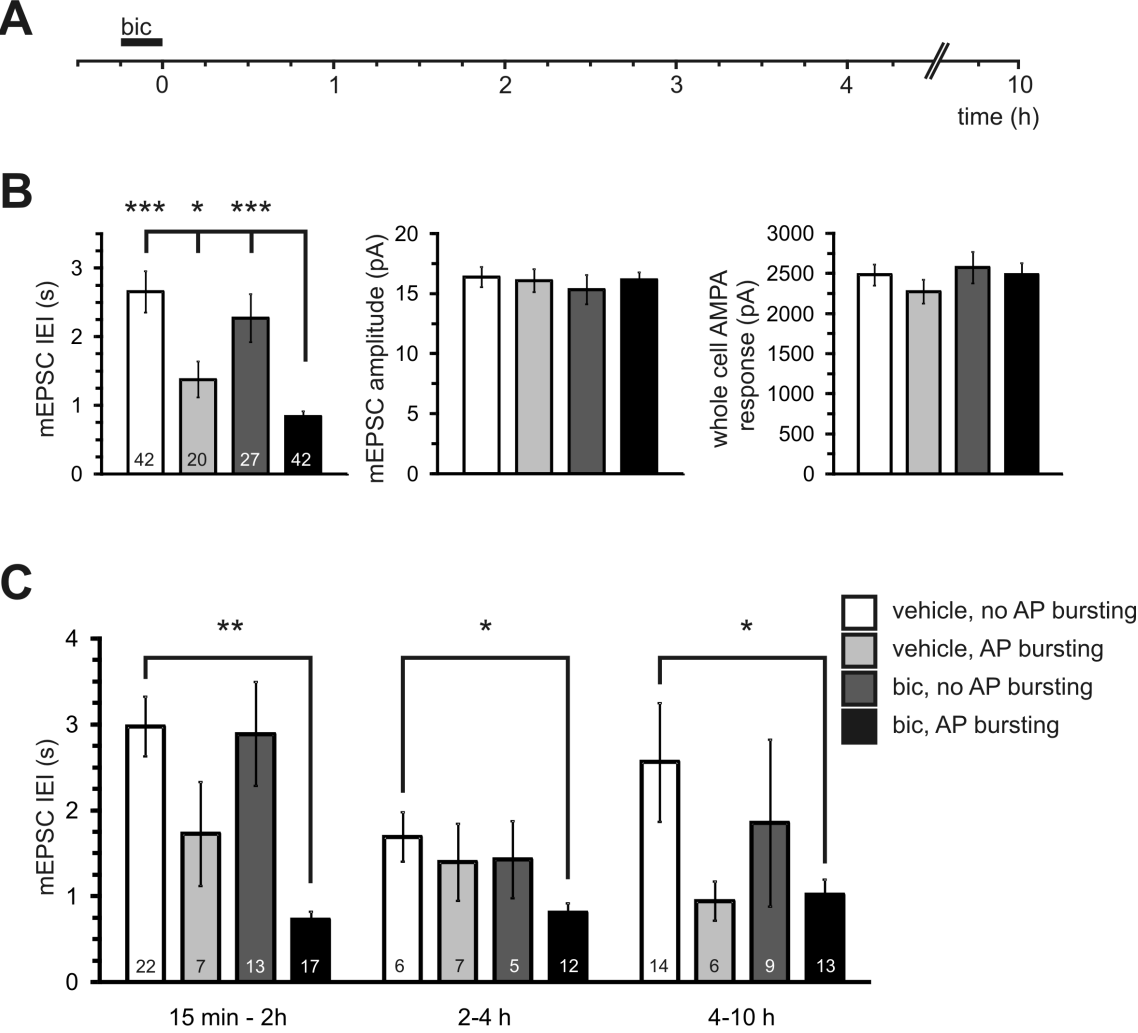
**Modulation of synaptic transmission over 10 hours after induction of AP bursting**. **(A) **Stimulation protocol used to induce action potential bursting (see Methods). Bicuculline (bic, 50 μM) or vehicle was applied for 15 minutes. mEPSCs were recorded after washout at various time points over the next 10 hours. **(B) **Mean values of mEPSC IEI (left), mEPSC amplitude (centre) and whole-cell AMPA response (right) recorded from cells treated for 15 minutes with either vehicle or bicuculline and exhibiting either AP bursting or random/silent activity patterns (see legend). Recordings span a 10 hour time period after treatment. Cell numbers in each group are shown in the left histogram. Asterisks indicate significant differences compared with the bicuculline treated, action potential bursting group. **(C) **The IEI data shown in B is separated into 3 groups to illustrate the time course of its modulation. Numbers within columns represent n of cells per group. * p < 0.05, ** p < 0.01, *** p < 0.001.

Synaptic potentiation has previously been demonstrated in hippocampal cultures at a single time point 30 minutes after a 15 minute induction period of AP bursting [18]. At this time point, mEPSC frequency was shown to be increased but little effect was seen on mEPSC amplitude or whole-cell AMPA responses. Although this study showed that AP bursting persists more than 6 hours after induction, it is not known whether mEPSC frequency remains increased and amplitude and AMPA responses remain unchanged at later time points. We first examined mEPSC results pooled from recordings at all time points within 10 hours after the 15 minute induction period. Spontaneous and in particular, bicuculline-induced AP bursting significantly reduced mEPSC IEI but did not significantly affect the mEPSC amplitude or the whole-cell AMPA current (Figure 2B). To better illustrate the time course of these effects, data was split into three groups: 15 min to 2 h, 2 to 4 h and 4 to 10 h. These groups span the period of transcription dependency of synaptic plasticity and AP bursting whereby the transcription inhibitor actinomycin D having little effect over the first 2 hours after induction, causes a slow return to baseline activity levels between 2 and 4 hours after induction [7,18]. Neither mEPSC amplitude nor whole-cell AMPA current was significantly affected by AP bursting at any time point (data not shown). However, when bursting and non-bursting cell groups were pooled, whole-cell AMPA response was slightly elevated in bicuculline vs. vehicle treated groups at the time points 15 min to 2 h (bicuculline: 2711 ± 175 pA; vehicle 2403 ± 136 pA; p < 0.05) and 2 h to 4 h (bicuculline: 2478 ± 198 pA; vehicle 2300 ± 139 pA; p < 0.05). No difference in AMPA whole-cell response was apparent in pooled data for the 4 to 10 h group. In contrast, IEI of mEPSCs was significantly shorter in comparison to untreated and vehicle treated cultures at all three time points (Figure 2C). Thus, a 15 minute induction of AP bursting induces long lasting changes in mEPSC IEI but not amplitude or whole-cell AMPA responses as was previously reported at a single 30 minute post-induction time point [18]. This indicates that, along with AP bursting, synaptic transmission is retained at an elevated level at transcription-dependent time points (i.e. > 2 h).

### AP bursting does not persist in all cells and is sensitive to actinomycin D

The persistence of bicuculline-induced bursting activity in hippocampal cultures and its sensitivity to the transcription inhibitor, actinomycin D has previously been investigated using arrays of planar extracellular electrodes, each of which can detect action potentials in multiple axons and somata within approximately 50 μm of the electrode [18]. The prevalence, persistence and transcription dependency of AP bursting in the post induction period has not been examined on a single cell level to date.

**Figure 3:**
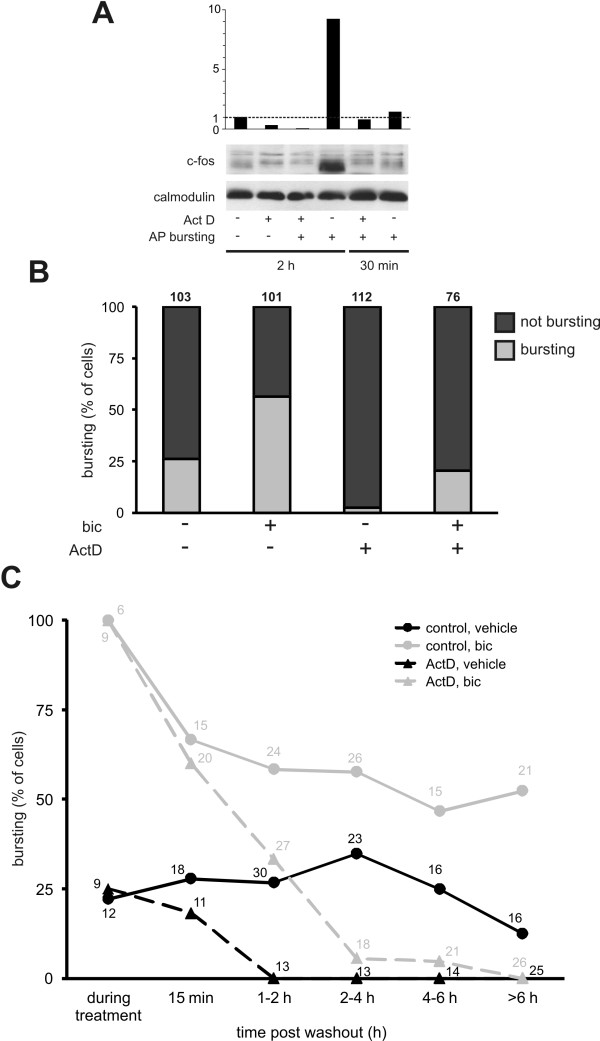
**The persistence but not the induction of AP bursting over the 10 hour post induction period is transcription-dependent**. **(A) **Western blot experiments showing the induction of the immediate-early gene c-fos either 2 hours or 30 minutes after a 15 minute period of bicuculline (50 μM) treatment in the presence or absence of actinomycin D (Act D, 8 μM). The histogram represents the intensities of the c-fos expression level relative to unstimulated control conditions. Endogenous calmodulin serves as loading control. **(B) **Histograms show the proportion of neurons exhibiting either bursting or random/silent activity patterns the 10 hours after a 15 min period of bicuculline or vehicle treatment in control and actinomycin D (ActD) treated neurons. Total neuron numbers in each group are indicated above each bar. **(C) **The bursting incidence data shown in B is separated into 6 groups to illustrate the time course of its modulation. Neuron numbers per group are indicated above each data point.

We first tested whether AP bursting-induced gene transcription is indeed inhibited by actinomycin D. Basal expression of the immediate-early gene c-fos in hippocampal cultures was very low in control conditions (Figure 3A, lane 1) and even lower in the presence of actinomycin D (Figure 3A, lane 2). Expression of c-fos was strongly induced 2 hours but not 30 minutes after induction of AP bursting with a 15 minute bicuculline treatment (Figure 3A, compare lanes 4 and 6). This induction of c-fos expression at the 2 h time point was abolished by the transcription inhibitor actinomycin D confirming that AP bursting-induced transcription is effectively blocked by this treatment.

The effects of bicuculline and actinomycin D treatment on AP bursting were next assessed on a single cell level 15 minutes to 10 hours after washout of bicuculline or vehicle. Bicuculline increased the overall incidence of AP bursting from 27 out of 103 neurons (26%) to 55 out of 101 neurons (54%) (Figure 3B). The proportion of cells displaying burst activity in either vehicle or bicuculline treated groups was considerably reduced however after exposure to actinomycin D (bursting in 2 out of 76 (3%) vehicle treated and in 23 out of 112 (21%) bicuculline treated neurons) (Figure 3B).

The time course of these effects is shown in Figure 3C. Bicuculline induced AP bursting in 100% of neurons regardless of any pre-treatment with actinomycin D. The proportion of neurons bursting 15 minutes after bicuculline washout was reduced to 67%, indicating that bursting activity is not maintained in all neurons but that this does not originate in a failure to participate in AP bursting during the induction period. However, this proportion declined only slightly over the next 6 hours to a level of 52%. Field recordings from MEAs have previously demonstrated that AP bursting persists for more than 6 hours in 90% of cultures [18]. This figure represents the persistence of bursting at a single electrode in a 60 electrode array rather than the number of individual neurons which maintain AP bursting.

Actinomycin D however, caused a sharp reduction in the incidence of AP bursting in the first 2 hours after its induction and had virtually abolished all bursting at later time points. This single-cell data supports previous evidence from multi-cellular recordings that bicuculline-induced AP bursting requires gene transcription for its maintenance and further reveals an inhibitory effect of actinomycin D on bursting of a spontaneous origin. Thus even spontaneous AP bursting requires ongoing gene transcription. The rapid shutdown of AP bursting in actinomycin D showed similar kinetics to the actinomycin D-induced decay of EPSC potentiation in acute slices [7] although somewhat faster than that reported by Arnold *et al*. (2005). The slower decay of potentiation in the MEA study is likely due to the quantification of spikes within bursts at a single electrode rather than sampling single cells across the network as we have done or assessing the amplitude of extracellularly recorded signals which reflects to some extent the number of responsive cells.

### The long-lasting, activity-induced increase in mEPSCs is actinomycin D sensitive

**Figure 4:**
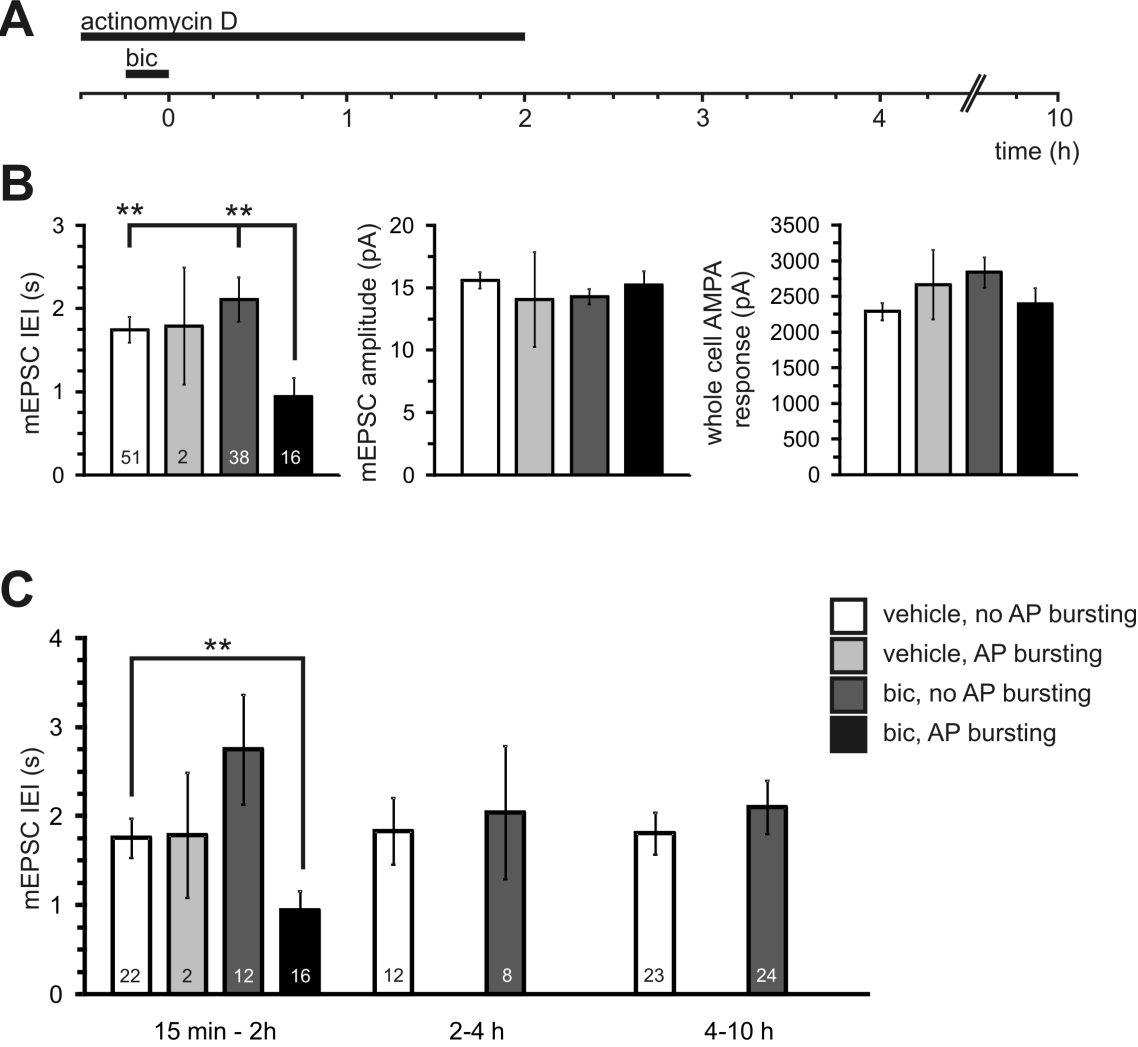
**AP bursting-induced reductions in mEPSC IEI are transcription-dependent**. **(A) **Stimulation protocol used to block gene transcription and to induce action potential bursting (see Methods). Actinomycin D (8 μM) was applied 15 minutes prior to bicuculline and left on the cells for 2 hours after bicuculline washout. The time line indicates the period over which mEPSCs were recorded after washout of bicuculline. **(B) **Shown are the mean values of mEPSC IEI (left), mEPSC amplitude (centre) and whole-cell AMPA response (right) recorded from cells treated with actinomycin D followed by a 15 minute treatment with either vehicle or bicuculline. Bursting activity was assessed at the beginning of each recording. Cell numbers in each group are indicated in the mEPSC IEI histogram. Asterisks indicate significant differences with respect to bicuculline treated, action potential bursting cells. **(C) **The IEI data shown in B is separated into 3 groups to illustrate the time course of its modulation by bicuculline after actinomycin D treatment. The numbers within columns represent n of cells per group. ** p < 0.01

Given the persistence of mEPSC upregulation at time points later than 4 hours after the induction of AP bursting and the dependence of AP bursting on gene transcription, we wished to directly test whether the long lasting upregulation of mEPSCs was transcription-dependent. mEPSCs were recorded from actinomycin D treated coverslips after the assessment of AP bursting at time points 15 minutes to 10 hours after bicuculline washout. In actinomycin D treated neurons as in control conditions (see Figure 2B), AP bursting did not significantly affect the mEPSC amplitude or the whole-cell AMPA current (Figure 4B). Actinomycin D did not affect the reduction in mEPSC IEI caused by AP bursting when data in each treatment group was pooled from all time points (Figure 4B). However, when this data from actinomycin D treated cells was separated into time groups, bursting and the reduction in IEI induced by AP bursting were only apparent at early time points (Figure 4C). At time points more than 2 hours after induction of AP bursting, actinomycin D had abolished both AP bursting and any differences in mEPSC IEI between vehicle and bicuculline treated groups. Thus, actinomycin D had abolished both AP bursting and the effects of AP bursting on mEPSC IEI at transcription-dependent time points (i.e. > 2 h) but not at early time points. We also noted that actinomycin D reduced mEPSC IEI in vehicle treated cells though this was only significant in the 15 min to 2 h time period (p < 0.01, compare Figure 2B with Figure 4B). Similar effects have been noted previously where the slope of field evoked EPSPs is increased during the first 2 hours of actinomycin D application [7]. Pharmacological blockade of bursting immediately after the induction-period returns mEPSC frequency to baseline.

**Figure 5:**
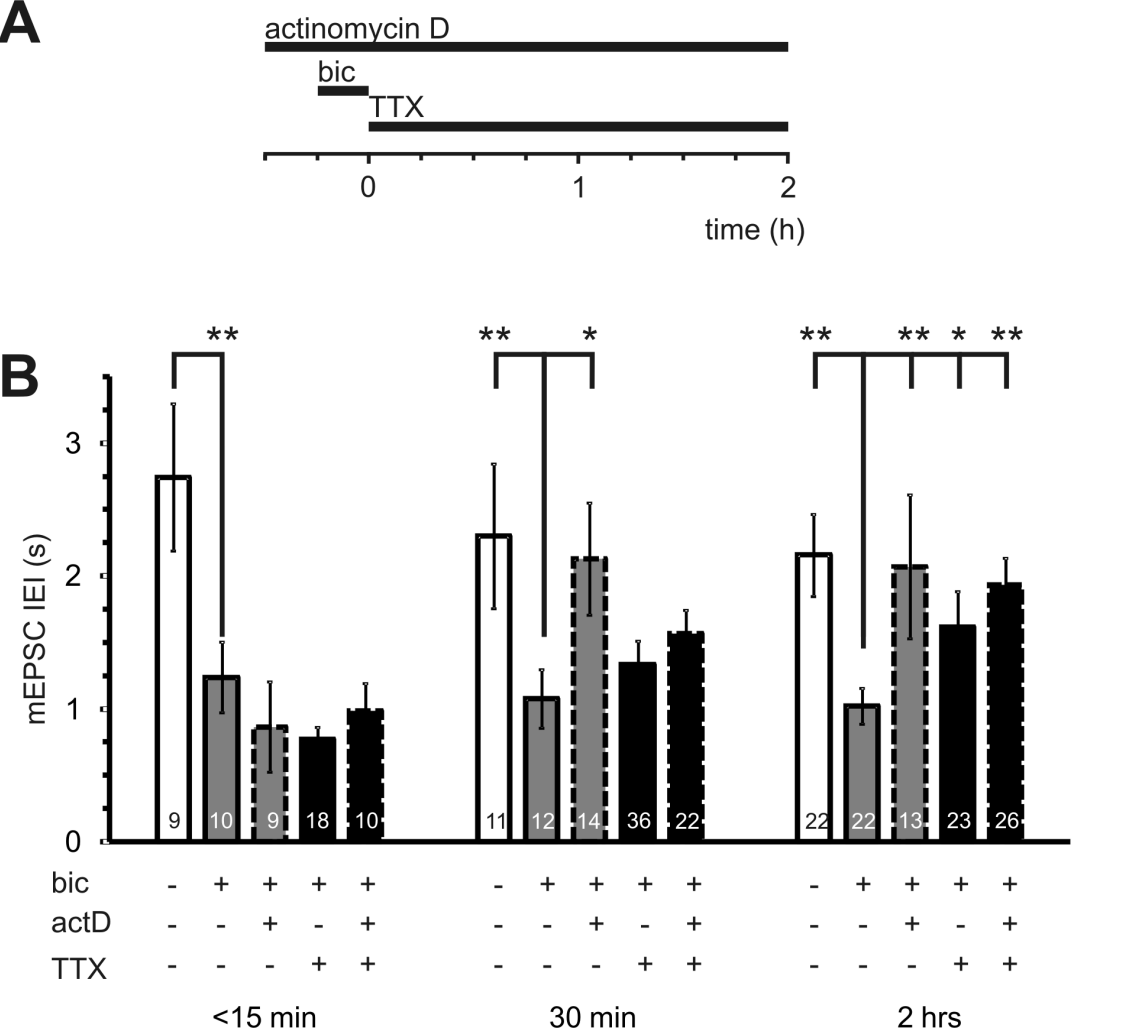
**The upregulation of mEPSCs does not persist if AP bursting is blocked immediately after the 15 minute induction period**. **(A) **Stimulation protocol used to block action potential bursting with TTX (1 μM) immediately after the 15 minute bicuculline application in presence or absence of actinomycin D (8 μM) which was applied 15 minutes prior to bicuculline and left on the cells for 2 hours after bicuculline washout (see Methods). The time line indicates the time window when mEPSCs were recorded after washout of bicuculline. **(B) **The histogram shows mean values of mEPSC IEI of vehicle (white) or bicuculline (grey or black) treated cells in the presence (dashed border) or absence (solid border) of actinomycin D that were either transferred to TTX (black) or not (grey) immediately after bicuculline washout. Data are separated into three groups to illustrate the time course of IEI-modulation by TTX application immediately after bicuculline stimulation. Numbers within columns represent n of cells per group. * p < 0.05, ** p < 0.01.

Given the correlation between mEPSC frequency and AP bursting and their parallel time course of inhibition by actinomycin D, we wished to establish whether mEPSC upregulation per se requires transcription or is just maintained by ongoing bursting which itself is transcription-dependent. We investigated whether a brief induction of AP bursting is sufficed to cause a long-lasting increase in synaptic transmission which persists even if AP bursting is blocked in the post induction period. Alternatively, if mEPSC upregulation were a short term effect of AP bursting then AP blockade would cause mEPSC frequency to rapidly decay back to baseline levels. To test these hypotheses, burst-activity was blocked immediately after the 15 minute induction period by TTX application before measuring mEPSCs within 15 minutes, 30 minutes or 2-3 hours. Since bursting was completely absent in all cells exposed to TTX in the post-induction period, data was pooled irrespective of burst activity prior to mEPSC measurement. A clear AP bursting-induced reduction in mEPSC IEI was still apparent at all time points in pooled control data (Figure 5B all bars without actinomycin D or TTX treatment). Pre-incubation with actinomycin D and/or TTX application after bicuculline washout did not disrupt the effects of AP bursting on mEPSC IEI within the first 15 minutes of the post-induction period (Fig 5B, see < 15 min time point). This result indicates that upregulation of synaptic transmission outlasts a brief blockade of AP bursting. However, blockade of AP bursting with TTX after the 15 minute induction period caused a gradual return of mEPSC IEI to baseline levels at the 2 h time point (Figure 5B, see 30 min and 2 h time points). This indicates that the activity-induced increase in mEPSCs requires either ongoing AP bursting or more than 15 minutes of AP bursting to persist until transcription-dependent, "late-phase" time points.

For direct comparison of the effects of actinomycin D, data from both bursting and non-bursting cells were pooled and re-plotted in Figure 5B. Actinomycin D treatment in the absence of TTX caused a more rapid loss of IEI potentiation at both the 30 min and 2 h time points. Surprisingly, the combination of both actinomycin D and TTX treatment in the post-induction period resulted in a less rapid decay of mEPSC IEI back to baseline levels at the 2 h time point. It is not clear why AP-blockade should slow the effects of actinomycin D in depotentiating synaptic transmission. One possibility is that the early phase of de-potentiation caused by actinomycin D is mediated by some activity-dependent depletion of the vesicular pool and may relate to the increased amplitude of evoked responses seen within 2 hours of actinomycin D application [7]. In summary, either ongoing activity or transcription or both are necessary for the potentiation of mEPSC frequency to persist beyond the 2 hour post-induction time point.

### A 2 hour critical period of activity is required to stabilise the increase in mEPSCs at the 6 hour time point

**Figure 6:**
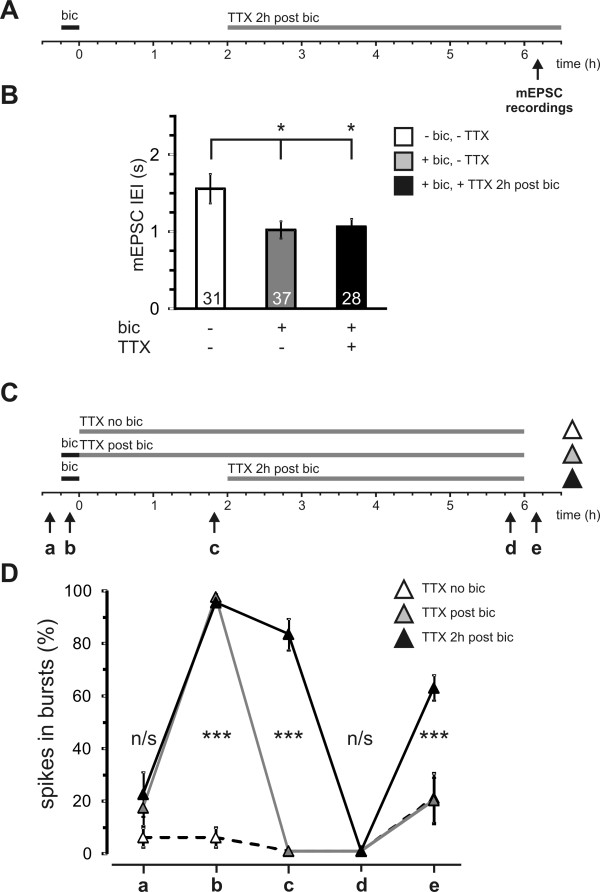
**Potentiation of mEPSC IEI enters an AP bursting-independent phase if spiking activity continues for some time after bicuculline treatment**. **(A) **Stimulation protocol used to block action potential bursting with TTX (1 μM) 2 hours after the 15 minute bicuculline application. The time line indicates the time window when mEPSCs were recorded after washout of TTX (see arrow). **(B) **The histogram shows mean values of mEPSC IEI of bicuculline or vehicle treated cells recorded 6 hours after the treatment (white and grey column). The black column represents cells that received a 4 hour TTX treatment commencing 2 hours after bicuculline washout. The numbers within columns represent n of cells per group. * p < 0.05 **(C) **Treatment protocols for the MEA-recordings shown in D used to block action potential bursting with TTX (1 μM) immediately (grey triangle) or 2 hours (black triangle) after the 15 minute bicuculline application. As a control, TTX was also applied immediately (white triangle) after vehicle treatment. Arrows indicate the time points when extracellular MEA-recordings were made. **(D) **The line graph represents the percentage of spikes within bursts in hippocampal cultures grown on MEAs. The neurons were treated as indicated in C and burst activity was measured for all three groups before, during, 2 hours after, 6 hours after bicuculline/vehicle treatment and after the final TTX-washout as indicated by the letters a-e. *** p < 0.001, n/s not significant.

We next tested the possibility that potentiated synaptic transmission requires continued AP bursting activity after the 15 minute induction period to become independent of AP bursting during the transcription-dependent late phase. Therefore, TTX was applied 2 hours after the 15 minute induction period leaving a time-window of 2 hours for the stabilisation of potentiated synaptic transmission. TTX was then applied for 4 hours before recording mEPSCs at the time point of 6 hours after the induction of AP bursting (Figure 6A). In control cells which did not receive TTX treatment, mEPSC IEI was significantly reduced in the bicuculline treated group compared to the vehicle treated group (Figure 6B). This reduced mEPSC IEI was also present in the group treated with TTX for 4 hours (Figure 6B). The amplitude of mEPSCs was unaffected by this 4 hour TTX-treatment indicating that synaptic scaling had not occurred (no TTX, no bic: 16.7 ± 0.99 pA; no TTX, bic: 15.2 ± 0.88 pA; TTX, bic: 16.4 ± 1.07 pA). Thus activity blockade for 4 hours does not affect the long-lasting, activity-induced increase in mEPSC frequency if AP bursting is allowed to proceed for the first 2 hours after induction. This shows that mEPSC upregulation is not a short term consequence of ongoing AP bursting but is a long lasting consequence of activity-induced synaptic potentiation.

### The reappearance of AP bursting is also sensitive to activity blockade during but not after a 2 hour critical period

We next asked whether AP bursting, like mEPSC upregulation, requires a 2 hour post-induction period for long-term stabilisation of a potentiated state at transcription-dependent time points (i.e., > 2 h). Since these experiments only required the assessment of burst activity, the use of multi-electrode arrays (MEAs) was favoured over single-cell patch clamp measurements. MEAs permit non-invasive, repeated assessments of the activity state at up to 60 electrodes across the culture. We used the same protocols shown in the patch clamp assessment of mEPSCs shown in Figures 5A and 6A to assess whether AP bursting reappears after reversible blockade of action potentials either in the first 2 hours or in the period between 2 and 6 hours after the induction period (Figure 6C). A control group without bicuculline treatment was also included to assess the effects of a 6 hour TTX application on activity after washout.

Quantification of bursting at the same electrodes at repeated time points is shown in Figure 6D. As has been reported previously [18], bicuculline treatment transformed low levels of baseline network activity into synchronous AP bursting upon the application of bicuculline. At the 2 hour time point synchronous AP bursting was maintained in the group which had not yet been treated with TTX and absent in the other two groups. Six hours after the induction period, prior to TTX-washout, no spike activity was present in any group. After TTX washout, AP bursting returned in networks where TTX was applied 2 hours after burst-induction but not in networks incubated with TTX immediately after bicuculline or vehicle treatment. Thus ongoing bursting during, but not after the 2 hour post-induction period is necessary for long-term maintenance of the potentiated network state.

In summary, a 15 minute induction of AP bursting establishes a potentiated synaptic and network state evident from an increased mEPSC frequency and AP bursting respectively. The persistence of this potentiated state in the first 2 hours does not require transcription but does require ongoing action potential activity. Persistence beyond the 2 hour time point requires both activity and transcription to have occurred in the first 2 hours but does not require ongoing activity thereafter. The direct link between transcription and a sustained increase in mEPSCs points to genes modulating quantal transmission in late-phase plasticity.

## Discussion

This study addresses for the first time the long-term persistence and transcription dependency of synaptic potentiation at the level of quantal synaptic transmission in single cells. We found that mEPSC frequency remains elevated for more than 6 hours after the induction of AP bursting in hippocampal cultures, an NMDA receptor-dependent model for synaptic activity-induced, long-lasting plasticity. This long-lasting potentiation of quantal transmission was transcription-dependent and required action potentials in the initial 2 hours after induction but was independent of ongoing activity thereafter. Thus, elevated quantal transmission is a direct consequence of gene transcription and is likely to be the mechanism which drives the network into a bursting state at transcription-dependent time points. This data identifies a functional consequence of the expression of genes previously identified by gene chip analysis as essential for the expression of long-lasting synaptic plasticity in this model.

### A new technique to investigate quantal transmission in late-phase plasticity

It has been known for 15 years that L-LTP is transcription-dependent and long lists of activity-induced genes have subsequently been published. However, the potential role of such genes in late-phase plasticity is obscured by a lack of knowledge about the mechanism of potentiated synaptic transmission in L-LTP. Minimal stimulation recordings have been used to assess quantal transmission in E-LTP but their application to L-LTP is impeded by the need for single-cell recordings over an extended time course of more than 3 hours. Our technique instead uses between-cell analysis based on brief recordings (< 20 min) which first begin after synaptic potentiation is established thus minimising the washout of intracellular messengers, a problem inherent with traditional whole-cell patch clamp recordings of L-LTP. mEPSC recordings also assess quantal transmission but unlike minimal stimulation experiments sample all synaptic connections rather than a potentiated subset thereof. Thus mEPSC analysis requires widespread synaptic potentiation which is presumably afforded by bicuculline which induced AP bursting in 100% of neurons in our hippocampal cultures. Nonetheless, large cell numbers are required to overcome high between-cell variability in mEPSC parameters arising from differences in the synaptic integration of neurons into the network. mEPSC analysis is often complemented by paired-pulse protocols, which detect changes in presynaptic release probability or coefficient of variation analysis which can estimate quantal content. However, such methods require recordings of evoked EPSCs which mostly evoke compound EPSCs in bursting cultures making them as yet technically unfeasible.

### The site of late-phase synaptic plasticity

Previous studies investigating quantal synaptic transmission at late time points have used activators or analogs of cAMP to induce chemical L-LTP in a large subset of synapses and have shown an increased action potential evoked presynaptic release [21] and mEPSC frequency but not amplitude [22]. In line with these results, we found an increased frequency of mEPSCs, with no change in amplitude. Such changes are mostly attributed to a higher release probability of presynaptic vesicles [23-27]. However, postsynaptic mechanisms can also increase mEPSC frequency without affecting mEPSC amplitude in a process termed synapse unsilencing [28]. To address this possibility we measured responses to bath application of AMPA to detect unsilencing mediated by *de novo *AMPA receptor insertion. Note that this method would not detect unsilencing mediated by the lateral diffusion of already functional AMPA receptors into the synaptic cleft. Previously, our group has shown a significant increase in AMPA response and mEPSC amplitude in within-cell but not between-cell analysis [18]. Here we observed a small increase in the whole-cell AMPA response at early time points but not at time points relevant for L-LTP. Therefore AP bursting appears to induce some *de novo *AMPA receptor insertion into the postsynaptic membrane which combined with the robust increase in mEPSCs observed would indicate either synapse formation or unsilencing. However, the fraction of this AMPA receptor pool is small and the variability of whole-cell AMPA-responses in between-cell experiments may preclude the detection of such subtle changes. Thus the side of the synaptic cleft at which potentiation of mEPSC transmission occurs cannot yet be clearly identified.

### Distinct mechanisms of early- and late-phase synaptic potentiation

Synaptic transmission was upregulated immediately after a 15 minute induction period of AP bursting and persists for 10 hours yet the mechanisms maintaining the increased frequency of mEPSCs do not remain constant over this timeframe. Ongoing action potential activity was necessary to maintain synaptic potentiation during the first 2 hours after induction but not thereafter. In contrast, gene transcription during the initial 2 hour critical period was necessary for the long-term but not the short-term maintenance of network potentiation. This distinguishes early and late phases of synaptic potentiation in this plasticity model which parallel those of E-LTP and L-LTP in acute brain slice experiments.

The dependence of early-phase potentiation on ongoing activity suggests that early-phase potentiation is a short-term consequence of ongoing burst-activity through mechanisms such as post-tetanic potentiation. In addition, our results reveal that activity, in the form of action potentials, is necessary during the initial 2 hours for the late-phase persistence of both AP bursting and increased mEPSC frequency. This suggests that activity plays a permissive role for the stabilisation of potentiated synaptic transmission. For example, the targeting or expression of mRNA transcribed during the initial 2 hour period may require ongoing activity [11]. After the initial 2 hour period, the potentiation of mEPSCs becomes permanently stabilised as a direct result of gene transcription.

An increased mEPSC frequency but not amplitude has previously been reported in hippocampal cultures 10 minutes after application of D1/D5 agonists [29]. This effect was due to protein kinase A-mediated activation of dendritic translation leading to local GluR1 expression and unsilencing of synapses. Blockade of NMDA receptor-mediated mEPSCs is known to activate dendritic translation and GluR1 expression within a 3 hour timeframe [30].

The direct link between transcription and a sustained increase in mEPSCs points to the involvement of genes modulating quantal transmission in late-phase plasticity. Indeed it seems likely that mEPSC upregulation underlies the maintenance of AP bursting in the late phase, showing a parallel time course in their expression and their sensitivity to activity or transcription blockade. In line with this, previous work has shown that single mEPSCs can influence firing in electrically compact neurons [31] and a burst of mEPSCs can drive action potentials in hippocampal pyramidal neurons[32]. The identification of genes underlying the potentiation of quantal synaptic transmission will help to unravel the molecular mechanisms responsible for the long-term maintenance of synaptic plasticity.

### Candidate genes for late-phase synaptic potentiation

The transcription-dependent mechanism which mediates mEPSC upregulation and is triggered by AP bursting now remains to be discovered. Screening the population of genes that were upregulated during AP-bursting [10] reveals several candidate genes whose protein products can regulate synaptic transmission. Three gene candidates stand out as being upregulated by AP bursting in an NMDA receptor-dependant manner and are known to act presynaptically to upregulate vesicle fusion and neurotransmitter release. These are *syntaxin3*, *homer1 *and *synaptotagminIV*. Syntaxin 3 belongs to the family of SNARE-proteins involved in the regulation of vesicle-fusion. However, its function to date has only been described at ribbon synapses in the retina [33]. Homer 1a and c have been suggested to be involved in the regulation of glutamate release although the underlying mechanism is not known [34]. SynaptotagminIV is a presynaptically expressed protein involved in the regulation of transmitter release but which has been shown to impede synaptic potentiation and short-term memory in the hippocampus [35]. Investigating these genes in the light of synaptic potentiation in hippocampal networks is therefore of great interest.

Four postsynaptic genes which are also upregulated by AP bursting and have the potential to modulate quantal transmission are *ephrin B2, catenin, brain-derived-neurotrophic factor *(*bdnf*) and *arc/arg3.1 *[10]. Retrograde messengers such as nitric oxide (NO), arachidonic acid and cell adhesion molecules can account for increased efficacy of transmitter release at presynaptic terminals [15,36,37]. Ephrin B2 is a member of the ephrin family of cell adhesion molecules which together with their receptors are considered to modulate synaptic transmission by bidirectional signalling [38]. Catenin, which binds to the cell adhesion molecule cadherin is involved in the control of vesicle clustering at presynaptic terminals [39] indicating a role in the control of release probability. Also, BDNF can acutely modify synaptic efficacy in LTP [40,41] most likely through a pre-synaptic mechanism [37] such as enhancing neurotransmitter release [42]. Other AP bursting-induced genes such as a *rc/arg3.1 *and *ac8 *(encoding adenylate cyclase 8) have also been identified in L-LTP studies. *Arc/arg3.1 *is of special interest since it was shown that upregulation of this gene in the postsynaptic neuron is directly linked to the modulation of synaptic transmission [43] by regulating AMPA receptor internalisation [11,44]. This may directly affect synaptic transmission and thus underlie synaptic potentiation. Such identified candidate genes can now be individually manipulated to test for a causal role in the increased mEPSC frequency which occurs in late-phase, transcription-dependent plasticity induced by AP bursting in hippocampal cultures.

## Conclusion

Long-term memory is thought to involve gene transcription which alters synaptic efficacy for extended periods; though the link between genes and synaptic function specifically altered in late-phase plasticity remains unknown. We show that the frequency of quantal synaptic events is increased for over 6 hours by synaptic activity known to induce gene transcription-dependent and NMDA receptor-dependent hippocampal plasticity. This identifies a specific synaptic function which is altered by one or more gene candidates already identified by gene chip analysis in the same cellular model. This puts us one step closer to matching gene to function in late-phase plasticity.

## Methods

### Hippocampal Cell Culture

Hippocampal neurons from new-born Sprague Dawley rats were prepared as described [45] except that growth media was supplemented with B27 (Gibco/BRL or Invitrogen, San Diego, CA) and 3% rat serum. Neurons were plated onto 12 mm glass coverslips at a density between 400 and 600 cells per mm^2^. All stimulations and recordings were done after a culturing period of 10 to 12 days during which hippocampal neurons develop a rich network of processes, express functional NMDA-type and AMPA/kainate-type glutamate receptors, and form synaptic contacts [46-48].

### The induction of network bursting and drug application

Bursts of action potentials throughout the neuronal network were induced by treatment of the neurons with 50 μM bicuculline for 15 minutes [18,46]. Bicuculline was dissolved in DMSO which did not exceed a final concentration of 0.05%. Bicuculline and vehicle treatments were always performed in parallel and recordings from both groups were made on the same day over the entire post-treatment time frame (0 to 10 h). In experiments where actinomycin D was used, the drug was applied 15 minutes prior to the bicuculline application at a final concentration of 8 μM and was left on the cells until the mEPSC-recordings were performed but not for longer than 2 hours.

### Patch clamp recordings

Whole-cell patch clamp recordings were made at room temperature from cultured hippocampal neurons plated on coverslips after a culturing period of 10 to 12 days. Coverslips were secured with a platinum ring in a recording chamber (PM-1, Warner Instruments, Hamden, CT, USA) mounted on either an inverted microscope (DMIRB, Leitz, Germany) or a fixed-stage upright microscope (BX51WI, Olympus, Hamburg, Germany). Differential interference contrast optics, infrared illumination and a CCD camera (PCO, Visitron Systems, Puchheim, Germany or Photometrics Coolsnap HQ, Pleasanton, CA, USA) were used to view neurons on a computer monitor connected through a contrast enhancement unit (Argus, Hamamatsu, Herrsching am Ammersee, Germany) to a video monitor or using a software interface (Metamorph, Universal Imaging Systems, Downington PA, USA). Patch electrodes (3-4 MΩ) were made from borosilicate glass (1.5 mm, WPI, Sarasota, FL, USA) and filled with a potassium intracellular solution (containing in mM: K-gluconate 143, MgCl_2 _2, NaCl 5, HEPES 10, K_2_-phosphocreatine 10, Mg_2_-ATP 4, Na_3_-GTP 0.3; pH 7.35 with KOH). The extracellular solution was a salt-glucose-glycine solution containing (in mM) NaCl 140, KCl 5.3, MgCl_2 _1, CaCl_2 _2, HEPES 10, glycine 1, glucose 30, Na-pyruvate 0.5. Recordings were made with either a Multiclamp 700A or 700B amplifier, digitised through a Digidata 1322A A/D converter and acquired using pClamp (Axon Instruments, Union City, CA, USA). All membrane potentials have been corrected for the calculated junction potential of -14 mV (JPCalc program by Dr. Peter H. Barry).

mEPSCs were recorded in the presence of TTX (1 μM, Alomone, Jerusalem, Israel). All voltage clamp recordings were performed at a holding potential of -80 mV at which GABA_A_-mediated IPSCs were outward and slower EPSCs indicative of NMDA receptor-mediated EPSCs were not apparent. All remaining mEPSCs were AMPA receptor-mediated as confirmed by their complete blockade by 6,7-dinitroquinoxaline (DNQX, 10 μM, Tocris, n = 6). mEPSC events were detected using the software MiniAnalysis (Synaptosoft, Decatur, GA, USA) with a 5 pA amplitude threshold and all mEPSCs were verified visually. Events occurring less than 10 ms after the previous event were included in frequency but not amplitude analyses. Access (range: 10 - 28 MΩ) and membrane resistance (range: 150 - 650 MΩ) were monitored regularly during voltage clamp recordings and data was rejected if changes greater than 30% occurred. Cumulative probability histograms, generated from at least 300 mEPSCs, were examined for each recording. Mean values for mEPSC parameters between groups were analysed using ANOVA and a post-hoc Tukey's test. All results are presented as mean ± S.E.M. Data points that exceeded the mean values by more than two times the standard deviation were considered outliers and not included into data analysis. Cell numbers and statistical analyses are given in the figures and their legends.

### Microelectrode array recordings

Neurons were plated onto microelectrode array (MEA) dishes containing a grid of 60 planar electrodes (Multi Channel Systems, Reutlingen, Germany) at a density of about 500 cells per mm^2^. Recordings were acquired with an MEA-1060 amplifier board (10 Hz-3.5 kHz, gain 1200, sampling frequency 20 kHz, Multi Channel Systems, Reutlingen, Germany). Recordings were performed after a culturing period of 10 to 14 days. Before stimulation, network activity was recorded for three minutes. Recurrent synchronous network bursting was induced by treatment of the neurons with 50 μM bicuculline (see above). After another three minutes of recording, bicuculline was washed away by changing the medium three times. Cultures were put back into the incubator and three minutes recordings were repeatedly performed at different time points following the washout of bicuculline. TTX (1 μM, Sigma, St. Louis, MO, USA) was added either immediately or two hours after washout of bicuculline. To wash out TTX at the end of the experiment, medium was changed three times at intervals of 10 minutes. Networks were allowed to recover from TTX block for another 10 minutes before the final recording. Spikes were detected with the integrated spike detector of the MC_Rack software (Multi Channel Systems, Reutlingen, Germany). Burst analysis was done with Neuroexplorer (NEX Technologies, ). All results are given as mean ± SEM. (Statistical tests were done by comparing data sets from treated cultures to controls for each time point separately by an independent samples t-test.)

### Immunoblotting

Expression of c-fos was measured by immunoblotting using an antibody to the c-fos protein (rabbit polyclonal antibody; Santa Cruz Biotechnology, Inc., Santa Cruz, CA, USA) as described previously [46]. Immunoblot analysis of endogenous calmodulin (monoclonal mouse-anti calmodulin antibody, Upstate Biotechnology, Temecula, CA, USA) expression was used to control for protein loading.

## Abbreviations

AP: action potential; BDNF: brain-derived neurotrophic factor; DNQX: 6,7-dinitroquinoxaline; E-LTP: early LTP; ERK1/2: extracellular signal-regulated kinase 1/2; IEI: inter event interval; L-LTP: late LTP; LTP: long-term potentiation; MEA: microelectrode array; mEPSC: miniature EPSC; NO: nitric oxide.

## Authors' contributions

JSW, HB and CPB designed the study. JSW and FH gathered and analysed the data. JSW and CPB prepared the manuscript. All authors have read and approved of the final version of the manuscript.
